# Hematology and serum biochemistry reference intervals for the common opossum *Didelphis marsupialis*

**DOI:** 10.1007/s11259-025-10684-1

**Published:** 2025-03-01

**Authors:** Claudia P. Ceballos, Estefanía Aristizábal-Parra, Viviana E. Castillo-Vanegas

**Affiliations:** Grupo de investigación GAMMA, Escuela de Medicina Veterinaria, Facultad de Ciencias Agrarias, Universidad de Antioquia, Medellín, Colombia

**Keywords:** Blood, Life stage, Marsupials, Reproductive stage, Sex, Urban habitats

## Abstract

The common opossum, *Didelphis marsupialis*, is a neotropical and synanthropic marsupial common and widespread in Latin America. The strong human-opossum conflict that results in high numbers of individuals with health problems demands information on physiological parameters to be used in veterinary medicine practice. The aim of this study was to estimate the reference intervals (RI) of hematology and serum biochemistry for this species which are lacking and evaluate its variability. Out of the 61 apparently healthy wild opossums evaluated, we found significant variability in the RI´s associated sex, life stage, habitat, and the reproductive stage of females. Males had higher values of RBC and hemoglobin than females, but females had higher values of MCH than males. Juveniles had higher values of MCV and MCH, but adults had higher values RBC, WBC and neutrophils. In addition, rural opossums had higher values of hemoglobin, MCHC, MCH and platelets, but urban opossums had higher values of WBC, particularly neutrophils and lymphocytes. Opossums are exposed to many different stressors in urban settings, and further research is needed to understand these physiological responses to urbanization. Finally, lactating females had higher values of monocytes and basophils compared to non-lactating females, potentially providing passive immunity through the milk to the immature neonates in the marsupium.

## Introduction

To provide an adequate veterinary care to wildlife is important to have the appropriate equipment and resources, but also biological information on the species to be treated. The hematology and serum biochemistry reference intervals (RI) are of particular importance to evaluate if the laboratory tests of a patient are altered or normal (Quagliardi et al. 2024). This is the case of the common opossum, *Didelphis marsupialis* (Didelphidae), a neotropical marsupial of medium body size up to 3450 g of body weight and 109 cm of total body length (Ceballos et al. 2024). This Opossum has an omnivorous diet, including small animals, fruits, insects, which may vary with the habitat and the season of the year (Tardieu et al. 2019; Cordero-Rodríguez 2000). This species is widely distributed, from southern Mexico to northern Brazil and Bolivia, and has large populations, thus for these reasons it has been assessed as Least Concern (Astúa et al. 2021).

Reference intervals for common opossums are urgently needed. First, there is a strong human-opossum conflict in several countries of Latin America such as Mexico, Trinidad and Colombia (Tardieu et al. 2020; Castro-Salazar et al. 2024; Ceballos et al. 2024). In Antioquia, Colombia, high numbers of individuals enter to the wildlife health and rehabilitation centers every day (Ceballos et al. 2024). In 2020, the Corporación Autónoma Regional de las Cuencas de los Ríos Negro y Nare (CORNARE) received 568 mammals, of which 74% were common opossums. The causes of entries recorded were: 54% were orphan opossums whose mothers had died, 14% had physical traumas with fractures in skull, legs, ribs or tail, body burns, or electrocution, 8% were attacked by dogs, 6% had diseases, and the remaining 18% had other causes or there was no data (data provided by CORNARE in 2021). Thus, physiological parameters are needed for a more assertive veterinary care. Second, opossums are consumed by humans (Barros and de Aguiar-Azevedo 2014; Astúa et al. 2021), which is a potential public health risk because opossums are reservoir for zoonotic parasites such as *Leishmania* sp., *Trypanosoma cruzi*, and Tetratrichomonas (Barros and de Aguiar-Azevedo 2014; Gamboa-Osorio 2020; Hernández-Montoya et al. 2024).

Hematology and blood chemistry values have been reported for several Didelphidae marsupials, including *Didelphis virginiana* (Lewis 1975; Zepeda-Espinosa et al. 2019; ZIMS 2020), *D. albiventris* (Tarragona et al. 2011), *D. aurita* (Casagrande et al. 2009), and *Monodelphis domestica* (Evans et al. 2010), however this information is lacking for *D. marsupials*. Moreover, some of these studies, and others in Dasyuridae marsupials (Fancourt and Nicol 2018) report the variability of those values by sex and age/life stage, reproductive stage and season. Herein we provide population-based reference intervals of hematology and biochemistry of the common opossum, *Didelphis marsupialis*, from Antioquia, Colombia. To better understand the variability of these values we evaluated if there were differences associated to sex (males vs females), life stage (juveniles vs adults), habitat of origin (urban vs rural), and the reproductive stage of females (lactating vs non-lactating).

## Materials and methods

### Animals and inclusion criteria

The inclusion criteria were all clinically healthy wild opossums, males and females, all sizes, trapped in urban and rural locations of Antioquia. We considered healthy animals those alert, with bright eyes, nose without discharge, and healed wounds. Animals excluded were those with open or bleeding wounds, with a depressed behavior (too docile), or any clinical sign of disease.

Animals included in this study (*n* = 61) were captured with tomahawk traps in July and August 2021, and from May to December 2022 (Ceballos et al. 2024). Traps were baited at 6–8 pm with fresh pork viscera, but also occasionally with cat food, and cooked eggs, and checked the following day around 6 am. Sampling included 5 municipalities of the department of Antioquia, Colombia: Medellín, Guarne, Rionegro, La Estrella, and individuals from Santuario were handed out to this study by the regional environmental authority CORNARE (Table 1). Medellín is a large city heavily urbanized with a human population of 6,546 people/km^2^ (DANE 2018), while the other municipalities between 2160 and 380 people/km^2^ are considered rural habitats (Table 1).

**Table 1:** Number of wild-caught opossums included in this study discriminated by municipality of origin, life stage, sex, and type of habitat based on population density (people/km^2^). Population density taken from (Gobernación de Antioquia 2020)

Municipality	*n*	Adult	Juvenile	Female	Male	Population density	Habitat
Medellín	27	21	6	11	16	6,546	Urban
Guarne	29	20	9	12	17	380	Rural
La Estrella	1	1	0	1		2,158	Rural
Rionegro	1	1	0		1	722	Rural
Santuario	3	2	1	2	1	482	Rural
Total	61	45	16	26	35		

The data on the sex, life stage (juveniles below 800 g of body weight and adults above 800 g), and the habitat type (rural or urban) where it was captured was recorded in a database. The reproductive stage of females was also recorded as lactating if it had neonates in the marsupium, or non-lactating if it had no neonates in the marsupium. Animals were manipulated with leather gloves and no anesthesia was used. All individuals were measured from the tip of the snout to the tip of the tail along the dorsal median line with a metric tape, and weighted in a cloth bag with a spring scale. Using these two measurements we calculated a ratio-based body condition index (BCI) which might be useful to assess the health condition in future studies: BCI = natural log (ln) of body weight / ln of total body length (Labocha et al. 2014). Summary statistics were calculated with package EnvStats in R environment (Millard 2013; R Core Team 2024). We tested for statistical differences on the medians of body weight and total body length between sexes and life stages with non-parametric Wilcoxon´s signed-rank test which allows for comparisons of groups with different sample sizes and variances. Analyses were done with wilcox.test function in the stats base package in R environment (R Core Team 2024).

### Blood sampling and analysis

A sample of blood (up to 2 ml) was taken from the caudal vein of the tail as described in Williams-Newkirk and colleagues (2013) and using 2ml disposable syringes with 23 G × 25 mm needles. Samples were collected from physically contained opossums, and it was done at the site of capture. Blood was deposited in two tubes, one with EDTA as anticoagulant to obtain whole blood for hematological analyses, and the other tube was empty to obtain serum for biochemical analyses. Blood samples were refrigerated and sent to Vitalab Diagnóstico Veterinario laboratory to be processed within 6 to 12 h after blood collection. The estimated amount of time the opossums were held in the traps until the blood collection was ± 6 h if the activity peak of opossums occurs by midnight (Rodriguez et al. 2022).

Hematology analyses included: red blood count (RBC), packed cell volume or hematocrit (PCV), Hemoglobin, mean cell volume (MCV), mean corpuscular hemoglobin concentration (MCHC), mean corpuscular hemoglobin (MCH), white blood cell (WBC), and the differential count: neutrophils, lymphocytes, monocytes, eosinophils, basophils, and platelets. Whole blood was analyzed by the electrical impedance method in an automated analyzer ABX Micros ESV 60 (Horiba ABX SAS, Kyoto, Japan), and the differential white cells count was done by cytomorphology confirmation with optical microscopy (magnification 1,000x) after blood smears were stained with Wright’s dye.

Chemistry analyses included measurements of blood urea nitrogen (BUN), creatinine, creatine kinase (CK), aspartate aminotransferase (AST), gamma-glutamyl transferase (GGT), alanine aminotransferase (ALT), total protein, albumin and globulin. For these purposes blood was centrifuged (2000 rpm for 10 min) to obtain the serum, and measurements were done following kinetics/colorimetric standard methods on a semi-automated chemistry analyzer Mindray BA 88A (Mindray Bio-Medical Electronics, Shenzhen, China).

### Reference interval values for hematology and blood chemistry

We followed the guidelines for the de novo reference intervals in veterinary species of the American Society for Veterinary Clinical Pathology (ASVCP) (Friedrichs et al. 2012). Outliers were included in the analyses because all opossums were clinically examined and found apparently healthy. Also, we expect that the inclusion of all values reflects the natural variability of the population and avoid artificially narrow RI´s. General descriptive statistics were calculated (mean, median, minimum, maximum and standard deviation). Given that we used wild-caught opossums, that health was not readily confirmed, and that our sample size was low (< 120 samples), we calculated 95% reference intervals (RI), i.e., lower 2.55% and upper 97.5% reference limits, with Bootstrap which is a robust method (distribution-independent) (Friedrichs et al. 2012). Reference intervals for groups with sample size < 20 were not calculated (Friedrichs et al. 2012). Finally, we calculated 90% confidence intervals (90% CI) for both, the lower and upper limits of the RI, using bootstrapping as well. Analyses were done in stats base package in R environment (R Core Team 2024). The visualize the results, boxplots were plotted showing all the data points and the RI calculated in this study using ggplot2 and dplyr packages in R environment (Wickham 2016; Wickham et al 2023; R Core Team 2024).

### Variability by sex, life stage and habitat type

To better understand the variability of hematology and blood chemistry values we evaluated if there were differences between sex (males vs females), life stage (juveniles vs adults), and habitat type (urban vs rural), and the reproductive stage of females (lactating young in the marsupium, or not). We did not check for pregnancy. For this purpose, we compared the medians of each group with Wilcoxon´s tests. Analyses were done with stats base package in R environment (R Core Team 2024), and the statistical significance was set at *P* < 0.05.

## Results

### Animals captured and morphometry

Out of the 61 animals trapped, there were 26 females and 35 males; and 16 were juveniles and 45 were adults (Table 1). Out of the 26 females, 19 had no young in the marsupium (empty), and 7 had young in the marsupium (range = 2–6). In addition, 27 came from the city of Medellin and were considered urban, and the remaining 34 were from less populated municipalities and were considered rural individuals. Table 1 discriminate them by municipality, sex and life stage.

The average body weight of females = 1196.6 ± 599.7 g (range = 140—2000), and the average body weight of males = 1513.1 ± 805.7 g (range = 144—3240), but they were not statistically different (*P* > 0.05). The average of body length of females = 79.1 ± 18.1 cm (range = 42—103.5), and that of males = 85,7 cm ± 17.6 (range = 42.7—109.5), and again they were not statistically different (*P* > 0.05). In relation to life stage, the average body weight of juveniles = 466.37 ± 244.88 g range = 140– 800), while the average body weight of adults = 1735.52 ± 542.91 g (range = 890– 3240), and as expected they were statistically different (*P* < 0.001). In addition, the average body length of juveniles = 59.67 ± 11.87 cm (range = 42– 78.5), and the average body length of adults = 91.57 ± 10.58 cm (range = 66– 109.5), which were statistically different (*P* < 0.001) as well.

### Body condition index

The average ln—ln BCI = 1.593 (interval = 1.3107– 1.7627, St. Dev. = 0.1065). A linear model of these two measurements (ln body weight ~ ln total body length) show they were positively correlated as expected and adequately fitted the data (intercept = −5.8114; slope = 2.9211; F_1,57_ = 356.5; *P* < 0.001; adjusted R^2^ = 0.8597).

### Hematology reference intervals (RI) and variability

**Fig. 1:**
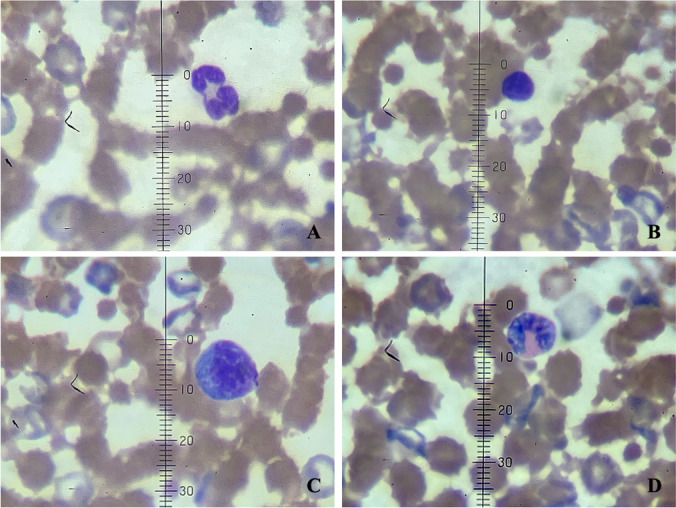
Microphotograph at 100X magnification of white blood cells of common opossum (*Didelphis marsupialis*) stained with Wright´s stain: **A**. Neutrophil (10 microns), **B**. Lymphocyte, **C**. Monocyte (12 microns), **D**. Eosinophil (11 microns)

**Fig. 2:**
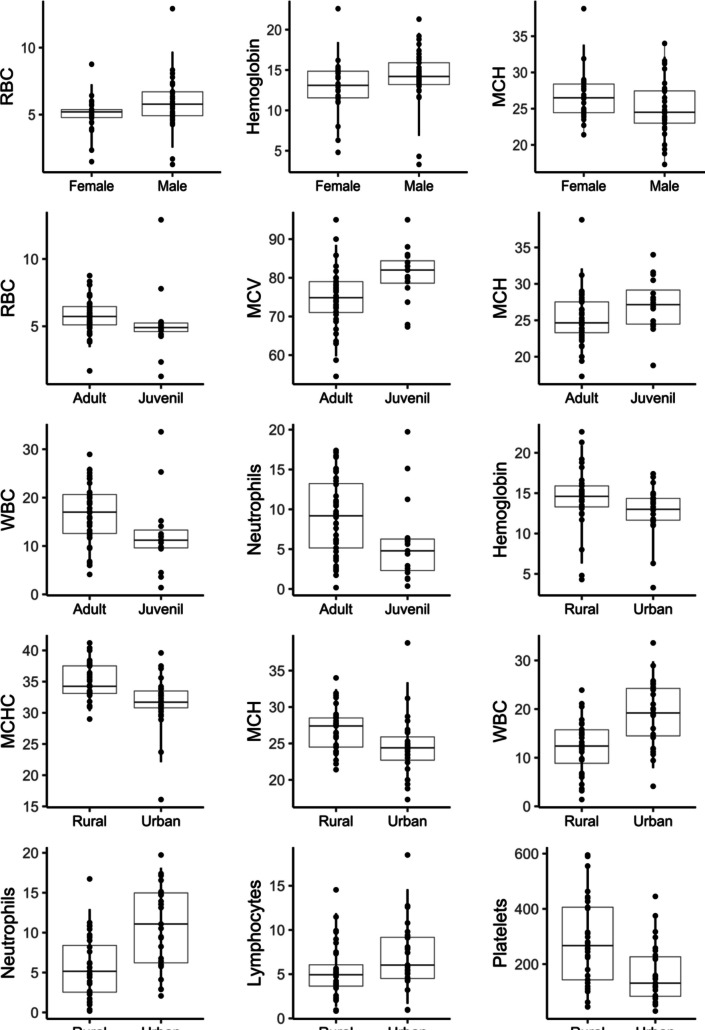
Boxplots of hematology reference intervals for wild common opossums (*Didelphis marsupialis*) discriminated by sex, life stage or habitat type (see Table 3). Boxplots show all data points, and vertical lines indicate de 90% RI calculated in this study

**Fig. 3:**
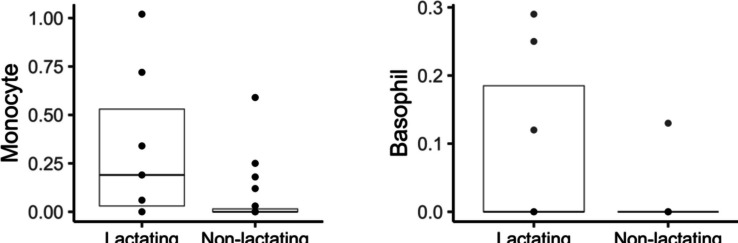
Boxplots of monocytes and basophils for wild common opossums (*Didelphis marsupialis*) in lactating vs non-lactating females (see Table 5). Boxplots show all data points (cero values overlap)

We report here the hematology RI’s for 61 wild common opossums captured in Antioquia Colombia (Table 2, Fig. 1). We found significant differences in some hematology (median) values associated to sex, life stage, type of habitat, and the lactating stage of females (Table 3, Figs. 2 and 3). Specifically, males had larger concentration of RBC (*P* < 0,05) than females. Also, hemoglobin was higher in males (*P* < 0.05), and females had higher values of MCH than males (*P* < 0.05), although the difference in the last two parameters was mild. Regarding life stage, adults had larger concentration of RBC (*P* < 0.05), WBC (*P* < 0.05), and neutrophils (*P* < 0.05) than juveniles, but on the other hand, juveniles had larger concentration of MCV (*P* < 0.01) and MCH (*P* < 0.01). Habitat type also explained important hematology variability. Rural opossums had higher significant values of hemoglobin (*P* < 0.05), MCHC (*P* < 0.001), MCH (*P* < 0.01), and platelets (*P* < 0.01) than urban opossums. On the other hand, urban opossums had higher WBC (*P* < 0.001), particularly neutrophils (*P* < 0.001) and lymphocytes (*P* < 0.01) compared to rural individuals. Finally, we found that the reproductive stage of females also affected two hematology parameters. Females with lactating young (*n* = 7) in the marsupium had higher values of monocytes (*P* < 0.05) and basophils (*P* < 0.05) compared to females without lactating young (*n* = 19). RI for groups with sample size < 20 were not calculated (Friedrichs et al. 2012).

**Table 2:** Hematology reference intervals (RI) and confidence intervals (CI) for the lower and upper reference limits (LRL and URL) for wild common opossums (*Didelphis marsupialis*). The *P* value indicates the distribution of the data (Dist.) as Gaussian (G) or non-Gaussian (NG)

Measurand	Units	*n*	Mean	SD	Median	Min	Max	*P*	Dist.	LRL of RI	URL of RI	CI of LRL	CI of URL
PCV	%	61	41.48	11.17	41.8	9	68.1	< 0.01	NG	14.74	61.71	9.2—29.37	57.25—68.1
RBC	10^6^/µL	61	5.54	1.74	5.31	1.28	12.91	< 0.001	NG	2	9.11	1.28—3.89	7.58—12.91
Hemoglobin	g/dL	61	13.68	3.43	14	3	22.6	< 0.001	NG	5.34	20.12	3.3—9.5	17.8—22.6
MCV	fL	61	76.46	7.98	77.3	55	95	> 0.05	G	60.64	91.48	54.5—65.15	85.9—95
MCHC	g/dL	60	33.38	3.95	33.2	16.10	41.2	< 0.001	NG	25.18	39.,97	16.1—29.6	38.25—41.2
MCH	pg	61	25.74	3.75	24.9	17.30	38.8	> 0.05	G	19.12	33.51	17.3—20.7	31.25—38.8
WBC conc	10^3^/µL	60	15.41	6.97	15.05	1.4	33.6	> 0.05	G	3.43	28.01	1.4—5.21	25.21—33.6
Neutrophil	10^3^/µL	60	8.1	5.14	6.59	0.17	19.73	< 0.05	NG	0.75	17.43	0.17—1.63	15.93—19.73
Lymphocyte	10^3^/µL	60	6.12	3.48	5.22	0.83	18.48	< 0.01	NG	1.1	13.97	0.83—2.13	11.15—18.48
Monocyte	10^3^/µL	60	0.15	0.34	0	0	2.06	< 0.001	NG	0	1.01	0—0	0.62—2.06
Eosinophil	10^3^/µL	60	1.01	0.66	0.96	0	2.87	> 0.05	G	0.04	2.38	0—0.16	1.94—2.87
Basophil	10^3^/µL	60	0.03	0.09	0	0	0.37	< 0.001	NG	0	0.30	0—0	0.21—0.37
Platelet	10^3^/µL	61	226.93	146.49	218	30	595	< 0.001	NG	45.91	543.19	30—59	454—595

*PCV* packed cell volume or hematocrit, *RBC* red blood cells, *MCV* mean cell volume, *MCHC* mean cell hemoglobin concentration, *MCH* mean cell hemoglobin, *WBC* white blood cells

**Table 3:** Variation of hematology reference intervals for wild common opossums (*Didelphis marsupialis*) by sex, life stage, habitat type and reproductive stage of females (lactating vs non-lactating). Reference intervals for juveniles, lactating and non-lactating females were not calculated because sample size < 20 (Friedrichs et al. 2012)

Measurand	Sex / Life stage / Habitat	*n*	Mean	SD	Median	Min	Max	LRL of RI	URL of RI	95% CI of LRL	95% CI of URL	Significance (Wilcoxon’s test)
RBC	Females	26	5.05	1.30	5.21	1.49	8.76	2.52	7.26	1.49—4.19	5.98—8.76	W = 311.5; *P* < 0.05
	Males	35	5.90	1.94	5.78	1.28	12.91	2.55	9.69	1.28—4.54	7.48—12.91	
Hemoglobin	Females	26	12.92	3.4	13.1	4.8	22.6	6.65	18.44	4.8—11	15.2—22.6	W = 320; *P* < 0.05
	Males	35	14.25	3.39	14.2	3.3	21.3	6.87	19.56	3.3—11.7	17.61—21.3	
MCH	Females	26	26.73	3.45	26.5	21.4	38.8	22.43	33.81	21.4—23.75	28.81—38.8	W = 591; *P* < 0.05
	Males	35	25.01	3.84	24.5	17.3	34	18.7	32.15	17.3—2	30.61—34	
RBC conc	Juveniles	16	5.19	2.48	4.91	1.28	12.91					W = 504; *P* < 0.05
	Adults	44	5.75	1.27	5.73	1.69	8.76	3.46	8.18	1.69—4.48	7.26—8.76	
MCV	Juveniles	16	80.94	7.05	82	67.3	95					W = 171; *P* < 0.01
	Adults	44	74.63	7.68	74.85	55	95	59.7	88.45	54.5—63.74	82.78—95	
MCH	Juveniles	16	27.09	3.73	27.15	18.8	34					W = 171; *P* < 0.01
	Adults	44	25.11	3.58	24.65	17.3	38.8	19.21	32.09	17.3—21.4	28.7—38.8	
WBC conc	Juveniles	15	12.43	8.06	11.2	1.4	33.6					W = 476; *P* < 0.05
	Adults	44	16.7	6.11	17	4.12	28.93	5.81	26.36	4.12—7.01	24.5—28.93	
Neutrophil	Juveniles	15	5.99	5.45	4.79	0.36	19.73					W = 455.5; *P* < 0.05
	Adults	44	8.98	4.8	9.19	0.17	17.39	1.7	16.86	0.17—2.72	5.12—17.39	
Hemoglobin	Rural	33	14.40	3.73	14.6	4.3	22.6	6.31	20.92	4.3—11.7	18.8—22.6	W = 604.5; *P* < 0.05
	Urban	27	12.81	2.93	13	3.3	17.4	6.62	16.75	3.3—11.07	15.30—17.4	
MCHC	Rural	32	34.88	2.97	34.25	29	41.2	30.38	40.30	29—31.64	38.53—41.2	W = 657.5; *P* < 0.001
	Urban	27	31.7	4.35	31.7	16.1	39.6	22.11	37.89	16.1—29.42	35.36—39.6	
MCH	Rural	33	26.83	3.02	27.4	21.4	34	22.26	32.41	21.4—23.58	30.78—34	W = 626.5; *P* < 0.01
	Urban	27	24.51	4.23	24.4	17.3	38.80	18.49	33.38	17.3—2	28.1—38.8	
WBC	Rural	32	12.06	5.46	12.4	1.4	23.9	2.96	21.45	1.4—5.37	17.8—23.9	W = 181.5; *P* < 0.001
	Urban	27	19.25	6.72	19.2	4.12	33.6	7.88	29.79	4.12—11.41	25.7—33.6	
Neutrophil	Rural	32	5.68	3.89	5.15	0.17	16.73	0.56	12.93	0.17—1.27	10.53—16.73	W = 189; *P* < 0.001
	Urban	27	10.84	5.14	11.08	2.07	19.73	2.81	18.11	2.07—4.75	16.53—19.73	
Lymphocyte	Rural	32	5.35	3.07	4.94	0.83	14.54	1.33	11.88	0.83—2.21	9.18—14.54	W = 300; *P* < 0.05
	Urban	27	7.03	3.82	6.03	0.94	18.48	1.66	14.60	0.94—3.22	10.78—18.48	
Platelet	Rural	33	280.33	157.7	267	45	595	56.87	564.20	45—97.2	470.2—595	W = 640.5; *P* < 0.01
	Urban	27	164.74	104.9	131	30	445	45.62	378.70	30—68.35	278.65—445	
Monocyte	Lactating	7	0.33	0.4	0.19	0	1.02					W = 31; *P* < 0.05
	Non-lact	19	0.06	0.14	0	0	0.59					
Basophil	Lactating	7	0.09	0.13	0	0	0.29					W = 41; *P* = 0.05
	Non-lact	19	0.01	0.03	0	0	0.13

### Serum biochemistry Reference Intervals (RI) and variability

**Fig. 4:**
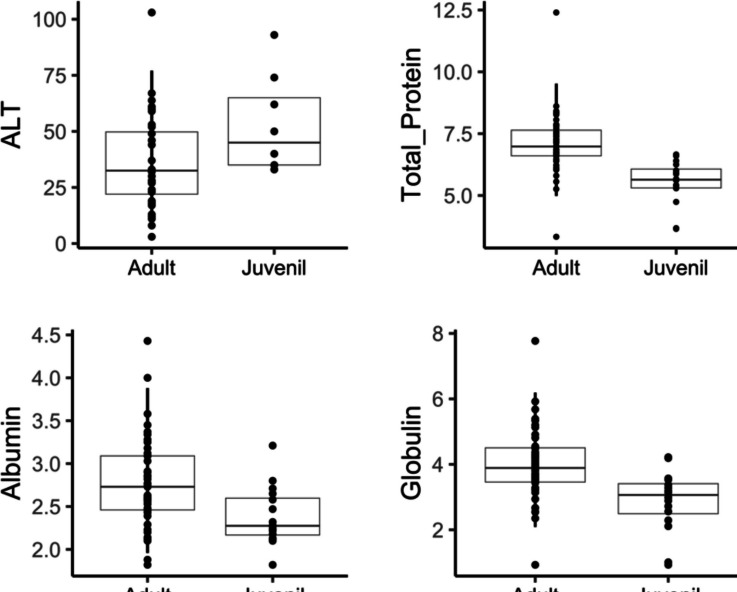
Boxplots of serum biochemistry for wild common opossums (*Didelphis marsupialis*) discriminated by life stage (see Table 5). Boxplots show all data points, and vertical lines of adults indicate de 90% RI calculated in this study

Serum chemistry RI’s for wild common opossums are listed in Table 4. We found no significant differences in the serum biochemistry associated to sex or habitat, but we did found differences in life stage (Table 5, Fig. 4). This is, adults had higher values of total protein (*P* < 0.0001), albumin (*P* < 0.01), and globulin (*P* < 0.001) compared to juveniles. Only ALT was found mildly higher in juveniles (*P* < 0.05).

**Table 4:** Serum biochemistry reference intervals for wild common opossums (*Didelphis marsupialis*). The *P* value indicates the distribution of the data (Dist.) as Gaussian (G) or non-Gaussian (NG)

Measurand	Units	n	Mean	SD	Median	Min	Max	*P*	Dist.	LRL of RI	URL of RI	95% CI of LRL	95% CI of URL
Urea nitrogen	mg/dL	43	46.28	25.42	40.33	12.63	122.21	< 0.001	NG	15.4	108.2	12.63—21.33	84.46—122.21
Creatinine	mg/dL	47	0.66	0.41	0.54	0.03	2.48	< 0.001	NG	0.2	1.6	0.03—0.35	1.20—2.48
AST	U/L	41	226.05	108.90	204	50	423	> 0.05	G	70.4	404.8	50—93.15	372—423
CK	U/L	33	1871.61	2128.11	1440	457	13138	< 0.001	NG	632	6401.6	457—831.42	2398.05—13138
GGT	U/L	33	13.78	7.72	12.06	2.78	35.24	> 0.05	G	3.9	28.7	2.78—6.14	24.22—35.24
ALT	U/L	41	40.60	22.38	35	3	103	> 0.05	G	8.5	86.3	3—13	66.75—103
Total protein	g/dL	60	6.61	1.44	6.68	3.07	12.40	< 0.001	NG	3.7	9.2	3.07—5	8.3—12.4
Albumin	g/dL	60	2.65	0.53	2.59	1.67	4.43	< 0.05	NG	1.9	3.8	1.67—2.1	3.36—4.43
Globulin	g/dL	60	3.64	1.22	3.62	0.93	7.77	> 0.05	G	1.2	6	0.93—2.2	5.27—7.77

**Table 5:** Serum biochemistry reference intervals for wild common opossums (*Didelphis marsupialis*) discriminated by life stage. Reference intervals for juveniles were not calculated because sample size < 20 (Friedrichs et al. 2012)

Measurand	Life stage	*n*	Mean	SD	Median	Min	Max	LRL of RI	URL of RI	95% CI of LRL	95% CI of URL	Significance (Wilcox test)
ALT	Juveniles	8	52.75	21.87	45	33	93					W = 69; *P* < 0.05
	Adults	32	36.74	21.5	32.5	3	103	7.61	77.05	3—12.25	61—103	
Total protein	Juveniles	16	5.53	0.89	5.64	3.65	6.66					W = 618; *P* < 0.001
	Adults	43	7.1	1.28	6.98	3.33	12.4	4.99	9.52	3.33—6.04	8.33—12.4	
Albumin	Juveniles	16	2.38	0.34	2.28	1.82	3.21					W = 508; *P* < 0.01
	Adults	43	2.77	0.53	2.73	1.82	4.43	1.96	3.88	1.82—2.14	3.38—4.43	
Globulin	Juveniles	16	2.87	0.94	3.07	0.93	4.22					W = 545.5; *P* < 0.001
	Adults	43	3.98	1.14	3.89	0.93	7.7	2.09	6.19	0.93—2.67	5.32—7.77

## Discussion

Overall, the RI´s for *D. marsupialis* in our study are wide but they encompass the RI reported in other species such as *D. virginiana* (Zepeda-Espinosa et al. 2019). For example, in the case of PCV, the 95% RI = 14.7– 61.7 in *D. marsupialis*, while for *D. virginiana* 90% RI = 31.4– 49.5 in females and 90% RI = 32.8– 56 in males. Other studies only report average values, for example in *D. virginiana* (Lewis 1975), and in *D. aurita* and *D. albiventris* (Casagrande et al. 2009), but overall, those average values are also within our RI in *D. marsupialis*. If needed, comparisons should be done carefully because of the different species, statistical methods and environmental conditions. The presented RI of *D. marsupialis* are specific to the conditions of this study, thus variations are expected (Friedrichs et al. 2012). Nevertheless, it is important to mention some tendencies in the variability of hematology and biochemistry values found in other species of *Didelphis* regarding to sex, life stage, habitat and reproductive stage.

Variability in hematology parameters may be explained by intrinsic factors such as sex, life stage/age, reproductive stage or body condition (Zepeda-Espinosa et al. 2019), or by extrinsic factors such as the season of the year (Tarragona et al. 2011; Beldomenico et al. 2008) or by health disruptors such as parasitic infections (Giacometti et al. 1972). Among the intrinsic factors, sex seem to affect few hematology values, or none at all, in *Didelphis* opossums. One study on *D. albiventris* in Argentina reported that neutrophils in females were doubled than those in males (Tarragona et al. 2011), and in our study on *D. marsupialis* red blood cells in males were about 10% higher than in females. However, two further reports on *D. aurita* and *D. albiventris* found no variation associated to sex (Casagrande et al. 2009; Tarragona et al. 2011). Neutrophils may increase during inflammatory responses or decrease with poor body condition, while higher number of red blood cells in males may be associated to heavier and larger males compared to females (Promislow 1991), as is the case of the population in this study (Ceballos et al. 2024).

Life stage is another intrinsic factor that seems to cause a more important variability in hematology compared to sex in *Didelphis* in general. For example, one study reported differences between juveniles and adults of *D. aurita* in RBC, hemoglobin, WBC, MCV, and MCHC (Casagrande et al. 2009). Similarly, there were also differences between juveniles and adults of *D. virginiana* in PCV, RBC, MCHC, MCH, lymphocytes, segmented neutrophils and platelets (Zepeda-Espinosa et al. 2019). Giacometti and colleagues (1972) also reported differences in life stage for some values of hematologic parameters in *D. virginiana* although no significance was reported.

The type of habitat is an extrinsic factor that was associated to a more throughout variability in hematology and biochemistry values, this is, between rural and urban opossums. This is important because urban opossums may be facing several urban stressors such as predator pressures by domestic animals and humans, artificial light and noise, roads that may be acting as barriers limiting their movements, pathogenic agents including parasites, and/or human aggressions (Fardell and Dickman 2023; Ceballos et al. 2024; Hernández-Montoya et al. 2024). We recently found that urban opossums in the metropolitan area of Valle de Aburrá (used in this study) are heavier and larger than rural individuals (Ceballos et al. 2024), and a potential explanation is that they may be eating non-natural food items (pet food or garbage, Hopkins and Forbes 1980; Cordero-Rodríguez 2000), that may have a deleterious effect on their health (Wist et al. 2022). On the other hand, opossums are successfully reproducing in this urban environment with an average litter size of 3.94 (interval = 2–7) (Ceballos et al. 2024). Thus, the extent to which these stressors in general affect “normal” values, or whether these values are physiological responses to changes in behavior, activity patterns, or diets as an adjustment to rapid urbanization (Schmidt et al. 2022; Rimbach et al. 2024) needs further studies.

Finally, the reproductive stage of females is an intrinsic factor that had an effect in some hematology values but not in the biochemistry, this is, lactating females had higher values of monocytes and basophils compared to non-lactating females. A potential explanation is that neonate marsupials are unable to respond immunologically right after birth because of the lack of mature lymphocytes, so they rely on the mother for immune defense through the milk (Stannard et al 2020). Further studies however are needed because these two types of immunological cells were not different between lactating vs non-lactating females of *D. virginiana* (Zepeda-Espinosa et al 2019).

The biochemistry parameters, on the other hand, are less variable than the hematology. In this study sex, habitat and reproductive stage of females did not affect the biochemistry, only life stage explained higher values of albumin, globulin and total proteins. Differences in total protein between juvenile and adult marsupials may be related to differences in the diet or body condition, but also diseases (Booth 2020). Urea, creatinine, AST, CK, ALT, and total protein reference values of *D. marsupialis* in this study include the average values reported in 6 individuals of *Didelphis virginiana* (Lewis 1975), however albumin was much higher in *D. marsupialis* than those reported for *D. virginiana* (Lewis 1975).

In conclusion, for the first time we report the hematology and serum biochemistry RI of the common opossum based on a wild population inhabiting in Antioquia, Colombia. We found variability of the RI associated to sex, life stage, reproductive stage of females, but mainly to the habitat type, i.e., rural vs urban habitats. Urban wildlife is growing around the world, and wildlife is adjusting to such new habitats, thus different physiological parameters, compared to their rural counterparts, should be expected. A final note of caution is that animals were apparently healthy, but we did not test for pathogens, except for parasites in the digestive system (Hernández-Montoya et al. 2024).
